# Human Bronchial Epithelial Cell Transcriptome Changes in Response to Serum from Patients with Different Status of Inflammation

**DOI:** 10.1007/s00408-024-00679-1

**Published:** 2024-03-17

**Authors:** Kokilavani Sivaraman, Bin Liu, Beatriz Martinez-Delgado, Julia Held, Manuela Büttner, Thomas Illig, Sonja Volland, Gema Gomez-Mariano, Nils Jedicke, Tetyana Yevsa, Tobias Welte, David S. DeLuca, Sabine Wrenger, Beata Olejnicka, Sabina Janciauskiene

**Affiliations:** 1 Department of Pulmonary and Infectious Diseases, Hannover Medical School, BREATH German Center for Lung Research (DZL), Feodor-Lynen-Str. 23, 30625 Hannover, Germany; 2 Department of Molecular Genetics, Institute of Health Carlos III, Institute for Rare Diseases Research, CIBER of Rare Diseases (CIBERER), Majadahonda, 28220 Madrid, Spain; 3 Hannover Medical School, Central Animal Facility, Hannover, Germany; 4 Hannover Medical School, Hannover Unified Biobank, Hannover, Germany; 5 Department of Gastroenterology, Hepatology and Endocrinology, Hannover Medical School, Hannover, Germany

**Keywords:** Immune hyper-activation, Acute lung inflammation, Chemokine/cytokine profile, Gene expression, RNA-seq

## Abstract

**Purpose:**

To investigate the transcriptome of human bronchial epithelial cells (HBEC) in response to serum from patients with different degrees of inflammation.

**Methods:**

Serum from 19 COVID-19 patients obtained from the Hannover Unified Biobank was used. At the time of sampling, 5 patients had a WHO Clinical Progression Scale (WHO-CPS) score of 9 (severe illness). The remaining 14 patients had a WHO-CPS of below 9 (range 1–7), and lower illness. Multiplex immunoassay was used to assess serum inflammatory markers. The culture medium of HBEC was supplemented with 2% of the patient’s serum, and the cells were cultured at 37 °C, 5% CO_2_ for 18 h. Subsequently, cellular RNA was used for RNA-Seq.

**Results:**

Patients with scores below 9 had significantly lower albumin and serum levels of E-selectin, IL-8, and MCP-1 than patients with scores of 9. Principal component analysis based on 500 “core genes” of RNA-seq segregated cells into two subsets: exposed to serum from 4 (I) and 15 (II) patients. Cells from a subset (I) treated with serum from 4 patients with a score of 9 showed 5566 differentially expressed genes of which 2793 were up- and 2773 downregulated in comparison with cells of subset II treated with serum from 14 patients with scores between 1 and 7 and one with score = 9. In subset I cells, a higher expression of *TLR4* and *CXCL8* but a lower *CDH1*, *ACE2*, and *HMOX1*, and greater effects on genes involved in metabolic regulation, cytoskeletal organization, and kinase activity pathways were observed.

**Conclusion:**

This simple model could be useful to characterize patient serum and epithelial cell properties.

**Supplementary Information:**

The online version contains supplementary material available at 10.1007/s00408-024-00679-1.

## Introduction

The incidence of acute and chronic respiratory diseases has been increasing worldwide. Respiratory diseases are associated with various comorbidities and risk factors, including genetics, exposure to adverse environmental factors, and aging [1]. The complicated pathogenesis of human respiratory diseases and the difficulties in simulating the real state of diseases experimentally limit our knowledge of the key mechanisms and molecules involved in pathological processes and therapeutic approaches.

The respiratory system is responsible for the exchange of oxygen and other gases with the external environment and comprises multiple epithelial, endothelial, and mesenchymal cell lineages [2]. These cells create not only a physical barrier but also a host defense system by expressing various receptors and producing inflammatory mediators. Therefore, an improved understanding of respiratory cell properties is essential. The latest clinical and experimental data provide new insights into human lung epithelial cell (HBEC) heterogeneity and its roles in acute and chronic lung inflammation. For instance, HBEC expresses pattern recognition receptors, such as Toll-like receptors (TLRs) and cytokine receptors, which allow cells to initiate an immune response [3].

Therefore, human HBEC are useful tools for studying various aspects of the pathophysiology of the pulmonary epithelium. In vitro 2D or 3D models with primary human HBEC are advantageous because they originate from the relevant host, express relevant host factors, and are of less ethical concern than animal models. In this study, for a model, we therefore selected primary human bronchial epithelial cells (HBECs) in 2D cultures and exposed them to sera from COVID-19 patients with different clinical severities.

SARS-CoV-2 and other coronaviruses infect the upper and lower respiratory tract. The control of viral spread depends on HBEC [4]. The HBEC activation and/or dysfunction can occur due to viral effects, as well as the release of inflammatory mediators, oxidative stress, and immune cell responses. For example, a characteristic feature of severe SARS-CoV-2 infection is increased blood levels of cytokines, particularly IL-1β, IL-6, and TNF-α, and chemokines such as IL-8 and MCP-1 [5]. The hyperactivated immune system together with cytokine/chemokine-induced exacerbated airway cell activation can cause severe lung injury [6–8]. It is well recognized that respiratory tract infections are associated with exacerbation of asthma or chronic obstructive pulmonary disease, and cause high morbidity and mortality in different age groups of individuals, even in those without underlying risk factors for respiratory diseases [9, 10].

The courses of infectious diseases such as COVID-19 vary from moderate to severe and fatal. Disease severity is assessed based on inflammatory biomarkers in liquid biopsies and clinical examinations using various scoring systems. The main aim of our study was to use a simple HBEC-based cell model to evaluate the effect of serum factors, which actively contribute to the formation of a pro- or anti-inflammatory environment. For this, we used sera from COVID-19 patients with different degrees of severity.

## Materials and Methods

### Patients and Biomaterial

Serum samples from 19 patients with COVID-19 were collected between November 2020 and September 2021. Disease severity and clinical outcomes were assessed using the WHO clinical progression scale (WHO-CPS) (Table 1). On the day of sample collection, five of the 19 COVID-19 patients had a WHO-CPS score of 9 (most severe disease, requiring mechanical ventilation pO_2_/FiO_2_ < 150 and vasopressors, dialysis, or ECMO). The remaining 14 patients had a WHO-CPS of < 9 (range 1–7). Samples were obtained from the Hannover Unified Biobank (HUB). Sample processing and storage were performed following the standard procedures of the HUB, as described by Kopfnagel et al. [11]. All patients included in this study signed a written consent. To establish the cellular model, we also used serum from 6 healthy age-matched donors [mean (SD) 52 (4) years]. The ethics committee of the Hannover Medical School (MHH, 9001_BO_K and MHH-6895) approved the sampling and analyses.

**Table 1:** WHO-CPS used to validate disease severity of COVID-19 patients (adopted from Marshall et al. [12])

Patient state	Descriptor	Score
Uninfected	Uninfected; no viral RNA detected	0
Ambulatory mild disease	Asymptomatic; viral RNA detected	1
	Symptomatic; independent	2
	Symptomatic; assistance needed	3
Hospitalized: moderate disease	Hospitalized, no oxygen therapy*	4
	Hospitalized; oxygen by mask or nasal prongs	5
Hospitalized: severe diseases	Hospitalized, oxygen by NV or high flow	6
	Intubation and mechanical ventilation pO_2_/FiO_2_ ≥ 150 or SpO_2_/FiO_2_ ≥ 200	7
	Mechanical ventilation pO_2_/FiO_2_ < 150 (SpO_2_/FiO_2_ < 200) or vasopressors	8
	Mechanical ventilation pO_2_/FiO_2_ < 150 and vasopressors, dialysis, or ECMO	9
Dead	Dead	10

*NV* noninvasive ventilation, *ECMO* extracorporeal membrane oxygenation

*Hospitalized for isolation alone

### Multiplex Immunoassay

Patient serum was analyzed using the Inflammation 20-Plex Human ProcartaPlex panel (Invitrogen, Thermofisher Scientific, Waltham, MA, USA) multiplex assay to detect GM-CSF, IFNα, IFNγ, IL-1α, IL-1β, IL-4, IL-6, IL-8, IL-10, IL-12p70, IL-13, IL-17A (CTLA-8), TNFα, IP-10 (CXCL10), MCP-1 (CCL2), MIP-1α (CCL3), MIP-1β (CCL4), ICAM-1, CD62E (E-selectin), and CD62P (P-selectin) according to the manufacturer’s protocol. Readings were carried out using the Luminex device Bio-Plex 200 (Bio-Rad, Hercules, CA, USA), which is compatible with Luminex xMAP fluorescent bead-based technology (Luminex, Austin, TX, USA).

### Cell Culture

Primary HBEC, isolated from the bronchial surface of healthy Caucasian male 62 years of age, was purchased from PromoCell (Lot: 458Z015, Promocell, Heidelberg, Germany). HBEC were cultured in Airway Epithelial Cell Growth Medium at 37 °C and 5% CO_2_. Cells of passage 4 were used in the experiments. Serum was added to HBEC (2% final) for 18 h. To avoid experimental bias, HBECs from the same passage were treated simultaneously with all individual serum samples. Cell-free supernatants of HBEC were collected for cytotoxicity assay and ELISA and cells were collected for gene expression analysis.

### Lactate Dehydrogenase (LDH) Cytotoxicity Assay

LDH release in cell-free supernatants was measured using the LDH Cytotoxicity Detection Kit (Roche, Basel, Switzerland) according to the manufacturer’s protocol and analyzed using an Infinite 200 Pro Microplate reader (Tecan, Männedorf, Switzerland).

### Trypan Blue Viability Assay

After culture for 18 h in growth medium or medium supplemented with 2% patient serum, HBECs were stained using 0.4% trypan blue solution (Invitrogen, Thermofisher Scientific, Waltham, Massachusetts, USA) and visualized microscopically using Leica DMIL LED (Leica Microsystems, Wetzlar, Germany).

### RNA Isolation, Reverse Transcription, and Quantitative Real-Time PCR

RNA was isolated using the RNeasy Mini Kit (Qiagen, Hilden, Germany). cDNA was synthesized using High-Capacity cDNA Reverse Transcription Kit (Applied Biosystems, Thermo Fisher Scientific, Waltham, MA, USA). Quantitative real-time PCR was performed using TaqMan gene expression assays (Table 2) and TaqMan Gene Expression Master Mix (Applied Biosystems) with the StepOnePlus Real-Time PCR System (Applied Biosystems), according to the manufacturer’s instructions. *POLR2A* was used as a housekeeping gene in the same run. The selection of *POLR2A* was validated by RNA-seq analysis. Measurements were performed in duplicates. Gene expression was calculated using the 2∆Ct method (Ct value of the target gene − Ct value of the reference gene).

**Table 2:** Taqman gene expression assays (ThermoFisher Scientific, Waltham, MA, USA)

Target	Assay ID
*ACE2*	Hs01085333_m1
*CDH1*	Hs01023895_m1
*CXCL8*	Hs00174103_m1
*FITM1*	Hs00416856_m1
*FITM2*	Hs00380930_m1
*HMOX1*	Hs01110250_m1
*IL1A*	Hs00174092_m1
*PECAM1*	Hs00169777_m1
*POLR2A*	Hs00172187_m1
*SERPINA*	Hs01097800_m1
*SERPINE*	Hs01126606_m1
*TLR2*	Hs01872448_S1
*TLR4*	Hs00152939_m1
*TMPRSS2*	Hs00237175_m1
*TNF*	Hs00174128_m1
*VEGFA*	Hs00900055_m1

### ELISA

Serum levels of hyaluronic acid and alpha1-antitrypsin (AAT) were measured using a Hyaluronan Duoset ELISA kit (R&D Systems, Minneapolis, MN, USA, assay detection range: 0.37–90 ng/ml) and Human Serpin A1 Duoset ELISA kit (R&D Systems, assay detection range: 0.125–8 ng/ml). Assays were performed according to the manufacturer’s instructions. For quantification of IL-8 in cell-free culture supernatants, Human IL-8/CXCL8 Duoset ELISA kit was used (R&D Systems, assay detection range 31.3–2000 pg/ml). Measurements were performed in duplicates.

### RNA Sequencing (RNA-seq) Analysis

RNA sequencing analysis was performed as described previously [13]. The quality of the total RNA was assessed using 1% agarose gels and by Agilent 2100 Bioanalyzer using Agilent RNA 6000 Nano Kit (Agilent, Santa Clara, CA, USA). RNA-Seq libraries were prepared from 200 ng of RNA from each sample using TruSeq Stranded mRNA Kit (Illumina Inc., San Diego, CA, USA) following the recommendations from the manufacturer. Sequencing was performed at the Genomics Service and the Bioinformatics Facility (Institute of Health Carlos III, ISCIII) on a NovaSeq 6000 sequencer (Illumina Inc.) using 100 base read lengths in paired-end mode analyzed the obtained RNA-Seq data. A quality control analysis was based on Fast QC v0.11.3 (http://www.bioinformatics.babraham.ac.uk/projects/fastqc/). Due to the limited amount of COVID-19 patient serum, we performed RNAseq analysis with one technical repeat per serum sample.

For the data analysis, normalization and differential expression analyses were performed on raw counts using the R package DESeq2 v1.32.0, with default settings. Differentially expressed genes (DEGs) were defined as those with an adjusted *p* value of < 0.05. Gene set enrichment analysis (GSEA) was performed on DEGs using the R package Enrichr Version [3.0] (W. Jawaid (New York, NY, USA). Significant gene ontology biological process (GO BP) terms and KEGG pathways were defined as the gene set results acquired using Enrichr with an adjusted *p* value of < 0.05. The normalized gene expression levels and DEG results were visualized using R and related packages, including ggplot2 Version [3.3.5] (H. Wickham et al. from Palo Alto, CA, USA) [14], ggrepel Version [0.9.1] (K. Slowikowski from Boston, MA, USA), and pheatmap Version [1.0.12] (R. Kolde from Tartu, Estonia) [15, 16].

### Statistical Analysis

Statistical analysis and graphical data presentation were performed using GraphPad Prism (Version 9.1.2 226). The Student’s *t test* was used to compare two sample means for one variable. When the Shapiro–Wilk normality test failed, the nonparametric Mann–Whitney *U* test was used. When more than two groups were involved in the comparison, one-way ANOVA was used. If the normality test passed, the data were presented as mean (SD). If the normality test failed, Kruskal–Wallis nonparametric one-way analysis followed by the Mann–Whitney rank-sum test, and the data were presented as median and interquartile range (IQR, 25th–75th percentile). Statistical significance was set at *p* < 0.05.

## Results

### Patient Demographics

Serum samples were retrospectively collected from clinically well-characterized, non-vaccinated COVID-19 patients (November 2020 to September 2021). The severity of SARS-CoV-2 infections was evaluated using the WHO-CPS (Table 1, adopted from Marshall et al. [12]). On the day of sample collection, 5 of the 19 patients had a WHO-CPS of 9 (most severe disease, requiring mechanical ventilation pO_2_/FiO_2_ < 150 and vasopressors, dialysis, or ECMO). The age, BMI, and comorbidities of these latter patients were not significantly different from those of the 14 patients with lower WHO-CPS (1 to 7). As shown in Table 3, significantly lower serum albumin levels (approximately 40%) were found in patients with a score of 9 than in those with a score < 9. Although not statistically significant, patients with a WHO-CPS of 9 (*n* = 5) had higher serum levels of AAT, CRP, ferritin, and hyaluronic acid than those with a WHO-CPS below 9 (*n* = 14) (Table 3).

**Table 3:** Characteristics of patient cohort

Variables	WHO-CPS = 9	WHO-CPS < 9	*p* value
Groups, *n* (%)	5 (26.3)	14 (73.7)	
Age, mean (SD)	61.4 (3.0)	61.6 (20.7)	0.9798
Gender (female/male)	1/4	7/7	
BMI	34 (27–43)	28 (27–32)	0.4773
Place of birth (Europe/Other/Unknown, n/n/n)	4/1/0	8/5/1	
Smoking status (Active/Never-/Ex-/Unknown, n/n/n/n)	0/0/0/5	1/6/3/4	
Vaccination status (Yes/No, n/n)	0/5	0/14	
Death due to COVID-19, n (%)	3 (15.8)	1 (5.3)	
Comorbidities (Yes/No/Unknown, n/n/n)			
Lung disease	0/2/3	0/10/4	
Diabetes	0/3/2	2/8/4	
Heart disease	0/1/4	2/7/5	
Adiposity	2/1/2	2/6/6	
Arterial hypertension	4/1/0	5/8/1	
Heart disease	0/1/4	2/7/5	
Kidney disease	0/5/0	4/9/1	
Liver disease	2/3/0	1/12/1	
Immunological disease (Vasculitis, diverticulitis)	0/5/0	2/12/0	
Pregnancy	0/5/0	3/11/0	
Organ transplantation	0/5/0	1/13/0	
Active tumor (Yes/No/in remission)	0/5/0	2/10/2	
Chronic therapy (Yes/No, n/n/n)			
Cortisone	0/5	2/12	
Immunosuppressive drugs	0/5	3/11	
ICU, *n* (%)	5 (26.3)	5 (26.3)	
Mechanical ventilation, *n* (%)	5 (26.3)	1 (5.3)	
ECMO, *n* (%)	4 (21.1)	0 (0.0)	
Vasopressors, *n* (%)	5 (26.3)	0 (0.0)	
Oxygen by NIV or high flow, *n* (%)	0 (0.0)	4 (21.1)	
Oxygen by mask or nasal prongs, *n* (%)	0 (0.0)	6 (31.6)	
Dialysis (Yes/No/Unknown, n/n/n)	2/3/0	0/13/1	
Anticoagulation (Yes/No/unknown, n/n/n)	3/0/2	8/3/3	
Steroids, *n* (%)	5 (26.3)	9 (47.4)	
Complications			
Renal failure	3 (15.8)	4 (21.1)	
Liver failure	2 (10.5)	0 (0.0)	
ARDS	5 (26.3)	3 (15.8)	
Clinical parameters			
AAT (mg/ml), n/mean (SD)	5/756 (169)	14/603 (141)	0.0620
Albumin (g/l), n/mean (SD)	5/18.0 (2.1)	7/30.9 (8.2)	**0.0069**
CRP (mg/l), n/median (IQR)	5/93 (85–169)	11/31 (11–138)	0.1149
D-Dimer (mg/l), n/median (IQR)	5/2.11 (1.50–3.86)	11/2.44 (1.32–7.43)	0.7223
Ferritin (µg/l), n/mean (SD)	5/1001 (218)	11/556 (508)	0.0848
Hyaluronic Acid (ng/ml), n/median (IQR)	5/117.7 (62.1–509.8)	14/75.7 (26.1–130.6)	0.2193
INR (ratio), n/median (IQR)	5/1.11 (0.97–1,18)	11/0.89 (0.86–1.02)	0.0545
Lipase (U/l), n/mean (SD)	5/45.8 (29.8)	9/41.6 (32.6)	0.8140
Leukocytes (10^3^/µl), mean (SD)	126 (61)	86 (36)	0.1170
Neutrophils (10^3^/µl), mean (SD)	1061 (961)	683 (241)	0.4335

Reference levels: Albumin (35–52 g/l), CRP (< 5 mg/l), D-Dimer (0–0.5 mg/l), ferritin (27–365 µg/l), INR (0.90—1.25 ratio), lipase (13–60 U/l). If the Shapiro–Wilk normality test was passed, variables were shown as mean (SD), and *p* values were calculated using a two-sided unpaired *t* test. If the normality test failed, variables were presented as medians (IQR), and *p* values were calculated using the Mann–Whitney test. Statistical significance was set at *p* < 0.05. The significant *p* values are highlighted in bold

*ARDS* acute respiratory distress syndrome, *CRP* C-reactive protein, *ECMO* extracorporeal membrane oxygenation, *ICU* intensive care unit, *INR* international normalized ratio (measure for the risk of thrombosis), *NIV* noninvasive ventilation, *WHO-CPS* WHO clinical progression scale

### Serum Cytokine/Chemokine Levels in COVID-19 Patients

**Fig. 1:**
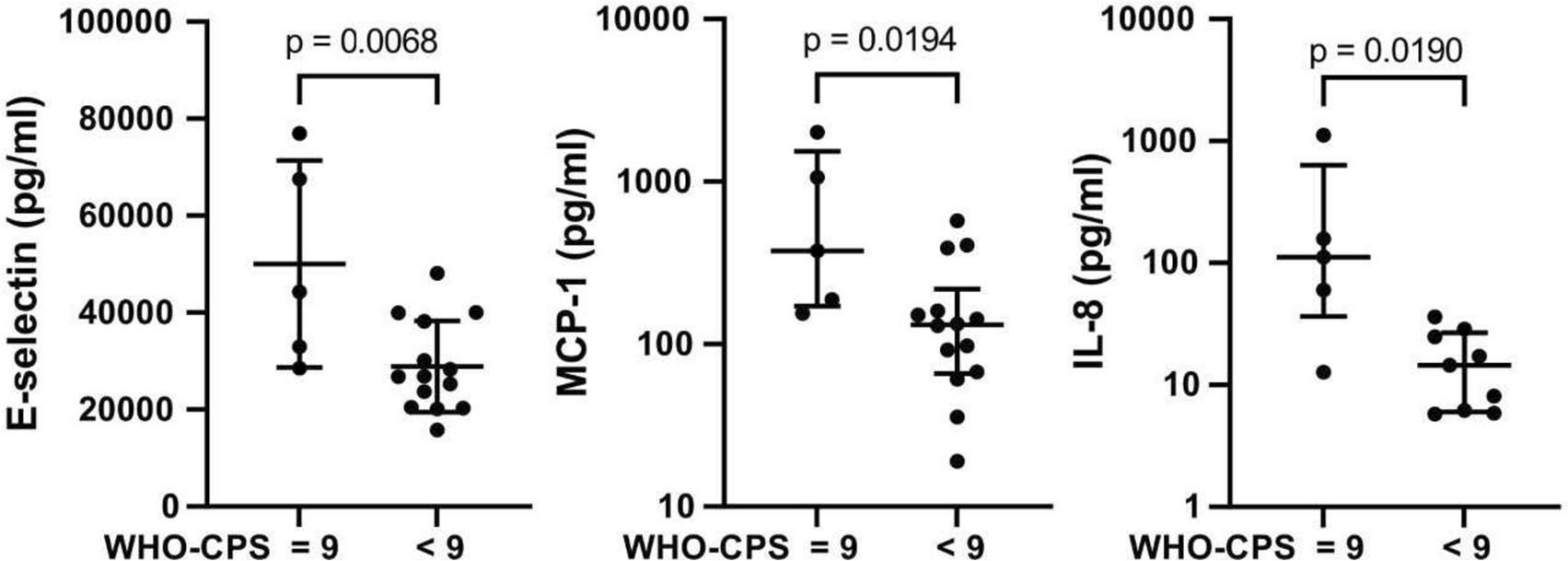
Serum levels of cytokines/chemokines. Serum samples from COVID-19 patients were analyzed using the Inflammation 20-Plex Human Multiplex Assay. All assays were performed in duplicate. If the Shapiro–Wilk normality test was passed, data are shown as mean (SD) and *p* values were calculated using a two-tailed unpaired t test. If the normality test fails, data are presented as median (IQR), and *p* values are calculated using the Mann–Whitney test. A *p* value below 0.05 was considered significant

Patient serum was analyzed using Inflammation 20-Plex Human Multiplex assay. Patients with a WHO-CPS score of 9 (*n* = 5) showed significantly higher levels of E-selectin, IL-8, and MCP-1 than those with a score < 9 (Fig. 1). Although the levels of the other markers did not differ statistically significantly between patient subgroups, the levels of most pro-inflammatory markers were higher in patients with a score of 9 (Supplementary Table 1). The GM-CSF, IFNγ, IL-1β, IL-4, IL-6, IL-10, and IL-13 levels were below the detection limits.

### Effects of Patient Serum on IL-8 Release and Inflammatory Gene Expression in HBEpC

**Fig. 2:**
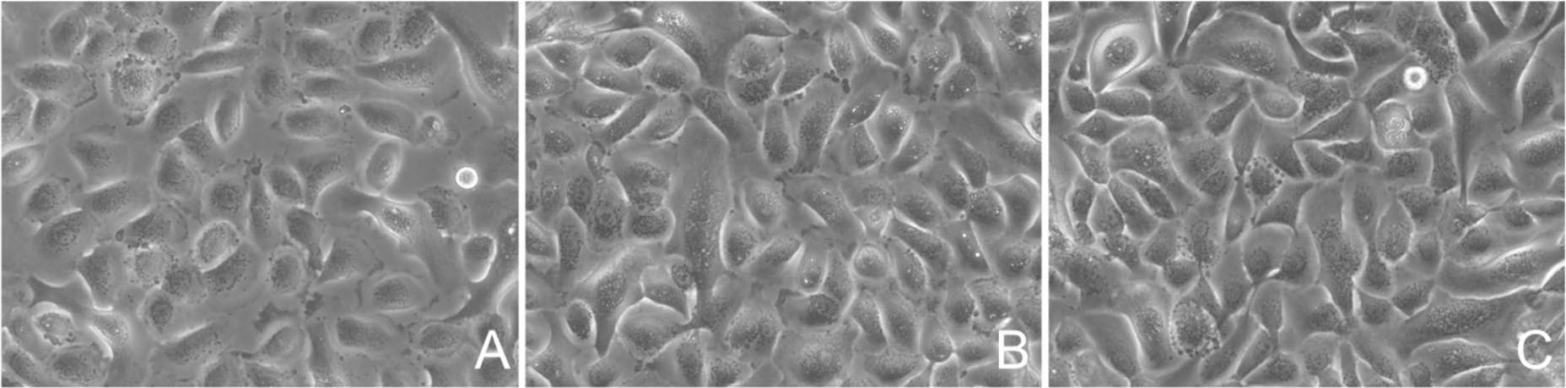
HBEpC morphology is unchanged in the presence of 2% COVID-19 patient serum. Representative images of cell morphology after 18 h culture in a medium supplemented with healthy donor serum (**A**), medium supplemented with 2% of serum from patient with WHO-CPS = 9 (**B**), and with serum from patient with WHO-CPS = 4 (**C**) were taken on a Leica DIML LED microscope equipped with camera using 10 × objective

Since serum profiles of COVID-19 patients were relatively similar, we then used a cellular model for a possible differentiation between patients with different disease severities. We incubated 2D HBEC cultures for 18 h in a medium supplemented with 2% healthy donor or each patient (*n* = 19) serum. A serum concentration of 2% was chosen based on preliminary experiments, which showed that this amount of serum did not alter cell viability or morphology (Fig. 2).

**Fig. 3:**
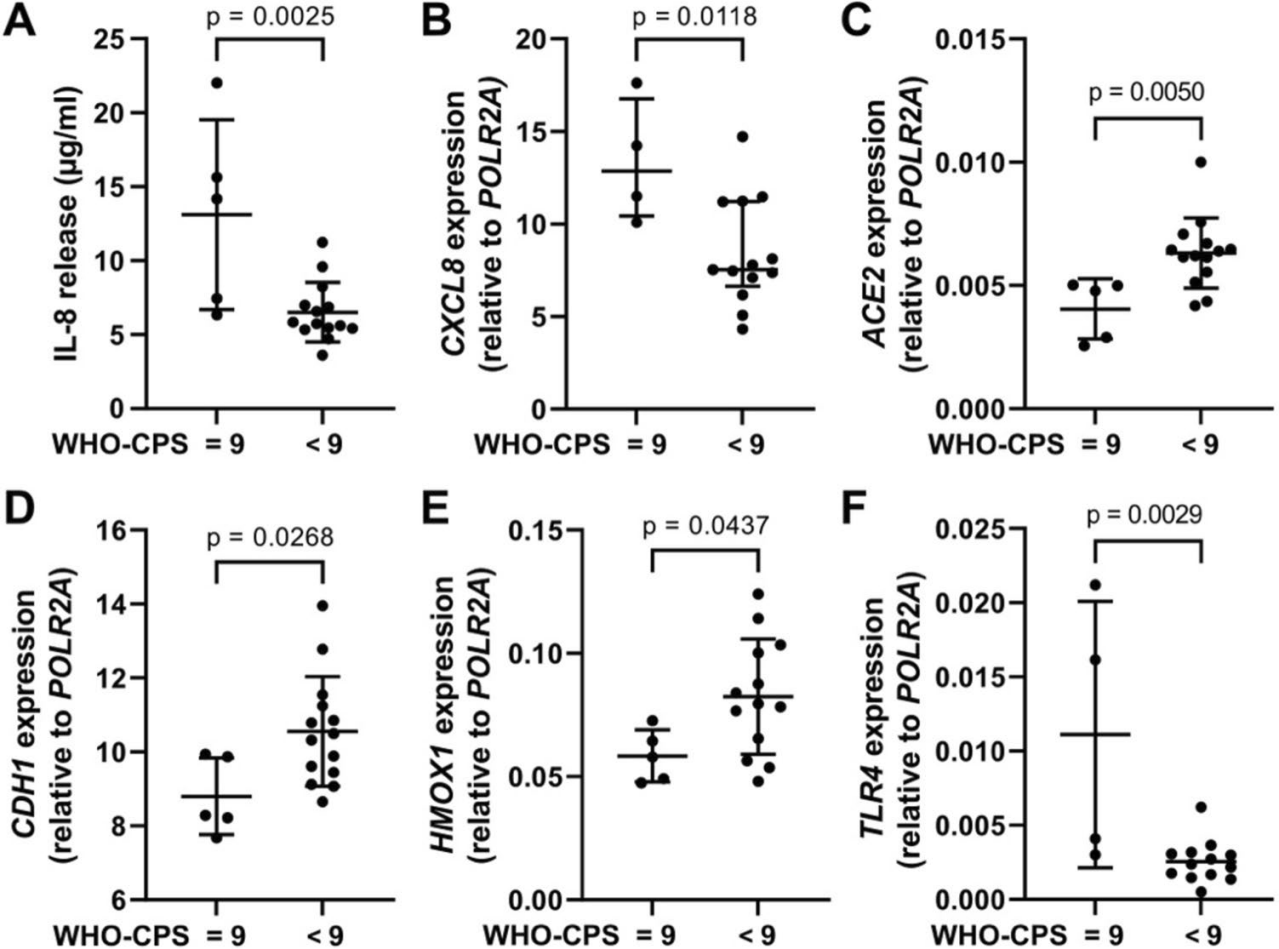
IL-8 release and the expression of specific genes in HBEC cultured in the presence of 2% patient serum (*n* = 19) for 18 h at 37 °C, 5% CO_2_. **A** IL-8 levels in cell supernatants supplemented with 2% patient serum after subtraction of IL-8 values detected in a cell-free cell culture medium supplemented with 2% serum. **B** to **F**. The RNA was isolated and analyzed by a real-time qPCR. All analyses were carried out in duplicates. If Shapiro–Wilk normality test passed, data are shown as mean (SD) and *p* values were calculated with two-sided unpaired *t* test. If normality test failed, data are presented as median (IQR) and *p* values are calculated with Mann–Whitney test. A *p* value below 0.05 was considered significant

The cells exposed to patient serum with WHO-CPS = 9 (*n* = 5) released higher levels of IL-8 and showed a higher *CXCL8* (IL-8 gene) and *TLR4* (Toll-like receptor 4) mRNA levels but significantly lower expression of *ACE2* (angiotensin-converting enzyme 2), *CDH1* (E-Cadherin), and *HMOX1* (heme oxygenase 1) than cells exposed to serum with WHO-CPS < 9 (*n* = 14) (Fig. 3). The expression of several other HBEC-related genes, selected to cover different signaling pathways, did not differ between cells treated with serum from patients with different WHO scores **(**Supplementary Table 2).

### Transcriptome Analysis of HBEC Cultured in Medium Supplemented with Patient Serum

**Fig. 4:**
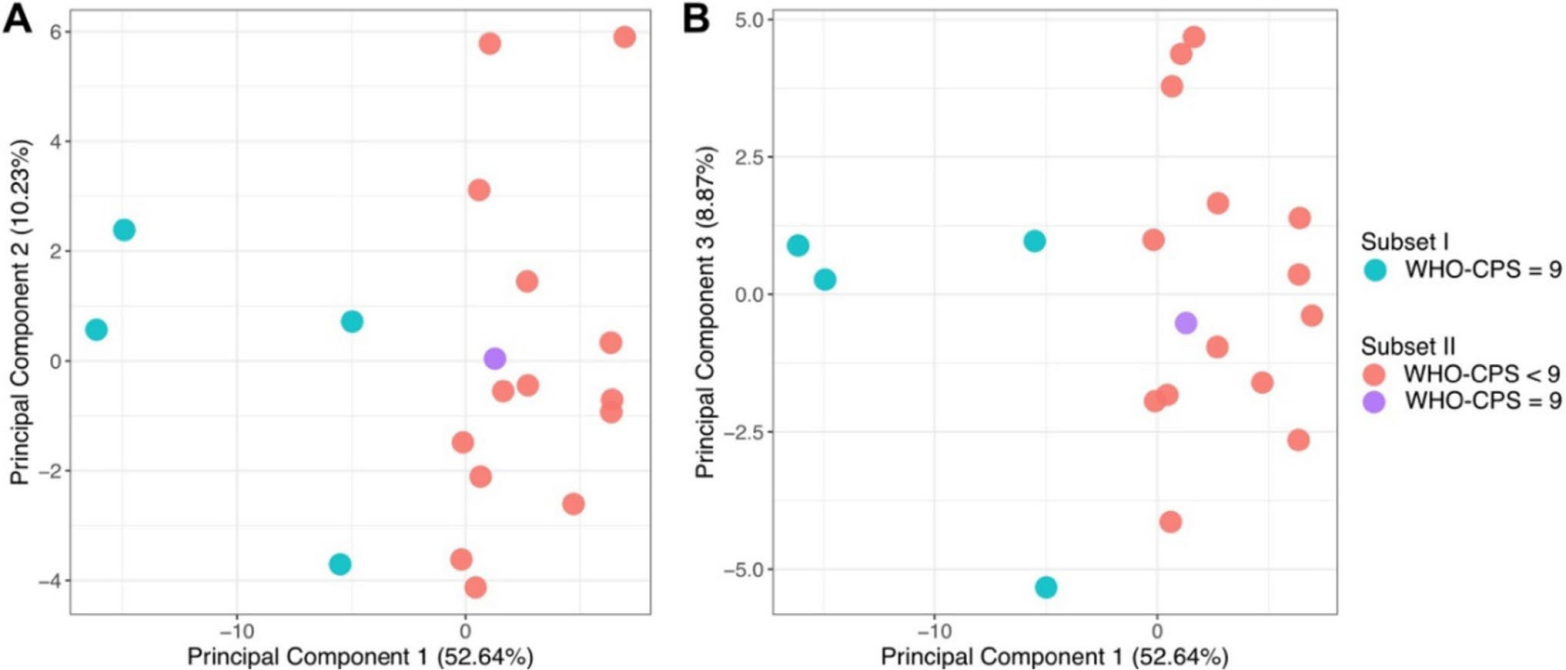
Principal component analysis of HBEC treated with 2% of patient serum and subjected to RNA-seq analysis. PCA on top 500 transcripts revealed a subset I of four clearly distinguishable patients all of which had a WHO-CPS of 9 (labeled in green). A subset II comprises 14 samples with a WHO-CPS of 1 to 7 (labeled in red) and one outlier sample with WHO-CPS = 9 (labeled in purple). The *x*- and *y*-axis represent the first (**A**) and second (**B**) principal components (PCs), respectively. The first PCA distinctly segregates the patients into two subgroups, accounting for 52.64% of the total variance among the samples

We performed a transcriptome data analysis (RNA-seq) of HBEC cultured in the presence of 2% serum from each patient using an unsupervised principal component analysis (PCA). To reduce the complexity caused by the high dimensionality of the original transcriptome data, we selected 500 transcripts with the highest variance, that is, the transcripts with the highest frequency in normalized read counts across all samples. The number of principal components (PCs) was set to ten. The PCA results showed that the first component could explain 52.64% of the total between sample variance, and the samples were divided into two subgroups along the *x*-axis representing this component (Fig. 4). All other nine components together accounted for less than half of the variance, with the second and third PCs accounting for only 10.23% and 8.87%, respectively (Fig. 4A, B). Therefore, PCA showed four clearly distinguishable cell samples, all treated with patient serum, classified as a WHO-CPS of 9. Cells treated with one of the serum samples with a WHO-CPS of nine did not occur in this subgroup.

Further RNA-seq analyses were performed on two cell subsets: first (I) treated with serum from four patients with score = 9, and second (II) treated with the serum of remaining patients, 14 with scores between 1 and 7 and one with score = 9. The characteristics of both subsets are shown in Supplementary Table 3. In general, cells in subset I showed 5566 DEGs, of which 2793 were upregulated and 2773 were downregulated compared with those in cell subset II. Heat maps show similarities and differences in the expression levels of the top 50 DEGs based on log2-fold change and -log10 adjusted *p* value across all 19 samples (Supplementary Fig. 1). In addition, the annotation of these genes to the Gene Ontology (GO) terms and related pathways analysis are presented in Supplementary Tables 4 and 5.

**Fig. 5:**
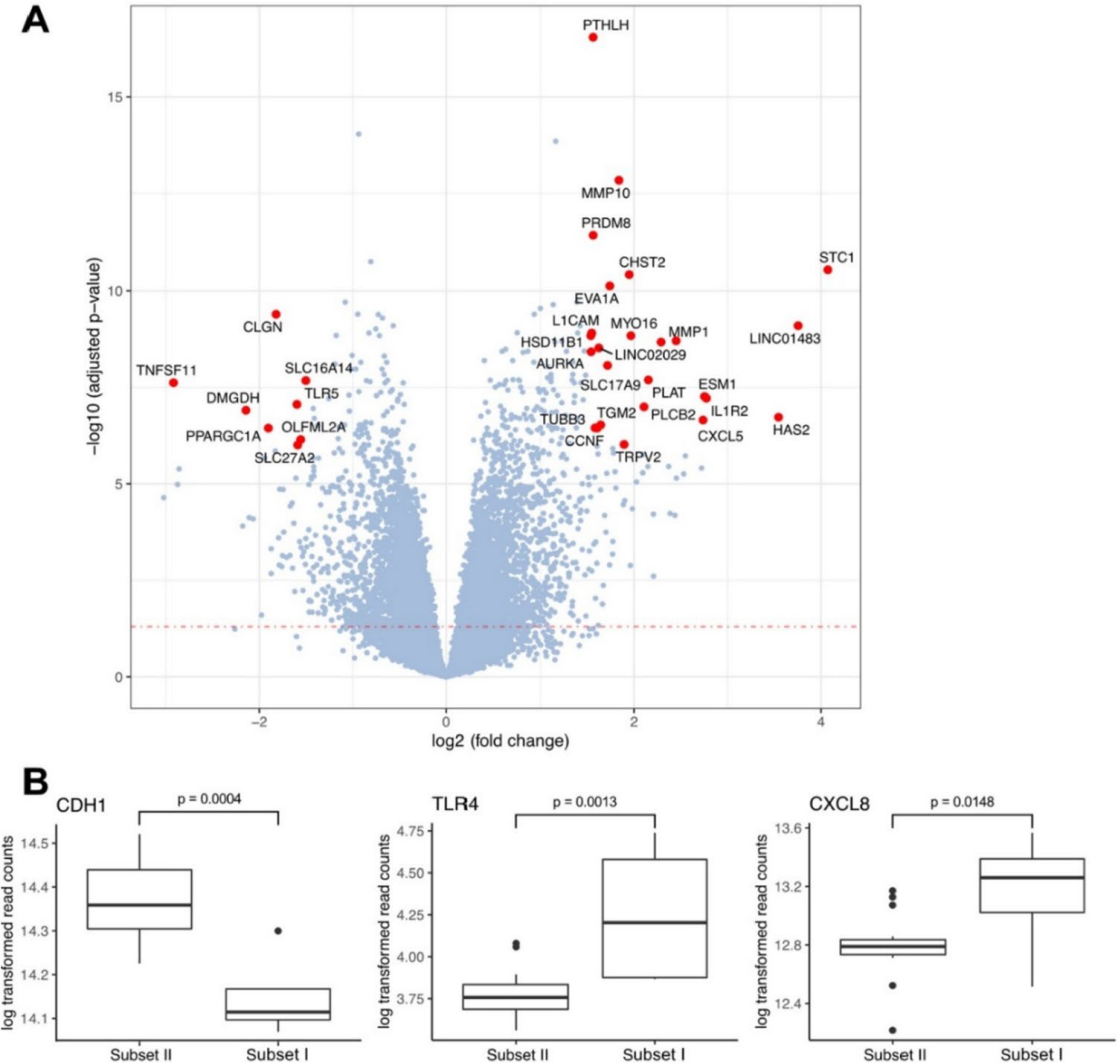
Volcano plot and normalized read counts of DEGs (*CDH1*, *TLR4* and *CXCL8*) identified by RNA-seq using DESeq2. **A** The volcano plot shows the differential gene expression analysis (DEA) of the HBEC cultured with 2% serum from SARS-CoV-2 infected 4 patients with a WHO-CPS = 9 (subset I), which were identified by PCA (Fig. 4, blue dots), compared to cells cultured with a serum from 15 patients (subset II, Fig. 4, red and purple dots). The *x*-axis denotes the log2-fold change, while the *y*-axis represents the -log10 transformed adjusted *p* values of the genes. Selected DEGs, possessing an absolute log2-fold change greater than 1.5 and adjusted *p* values less than 10^–6^, are highlighted in red and labeled. The red dotted line establishes a threshold of significance, corresponding to an adjusted *p* value of 0.05. **B** Box plots show DEGs identified by RNA-seq using DESeq2 in cells treated with serum of patient subsets I (Fig. 4, green dots, WHO-CPS = 9, *n* = 4) and II (Fig. 4, red and purple dots, *n* = 15)

To visualize the direction, magnitude, and importance of gene expression changes between cell subsets I and II, we generated a volcano plot and normalized read counts of DEGs (*CDH1, TLR4*, and *CXCL8*) identified by RNA-seq using DESeq2 (Fig. 5A, B). These genes were also identified as differentially expressed in the quantitative real-time PCR experiments (Fig. 3).

**Fig. 6:**
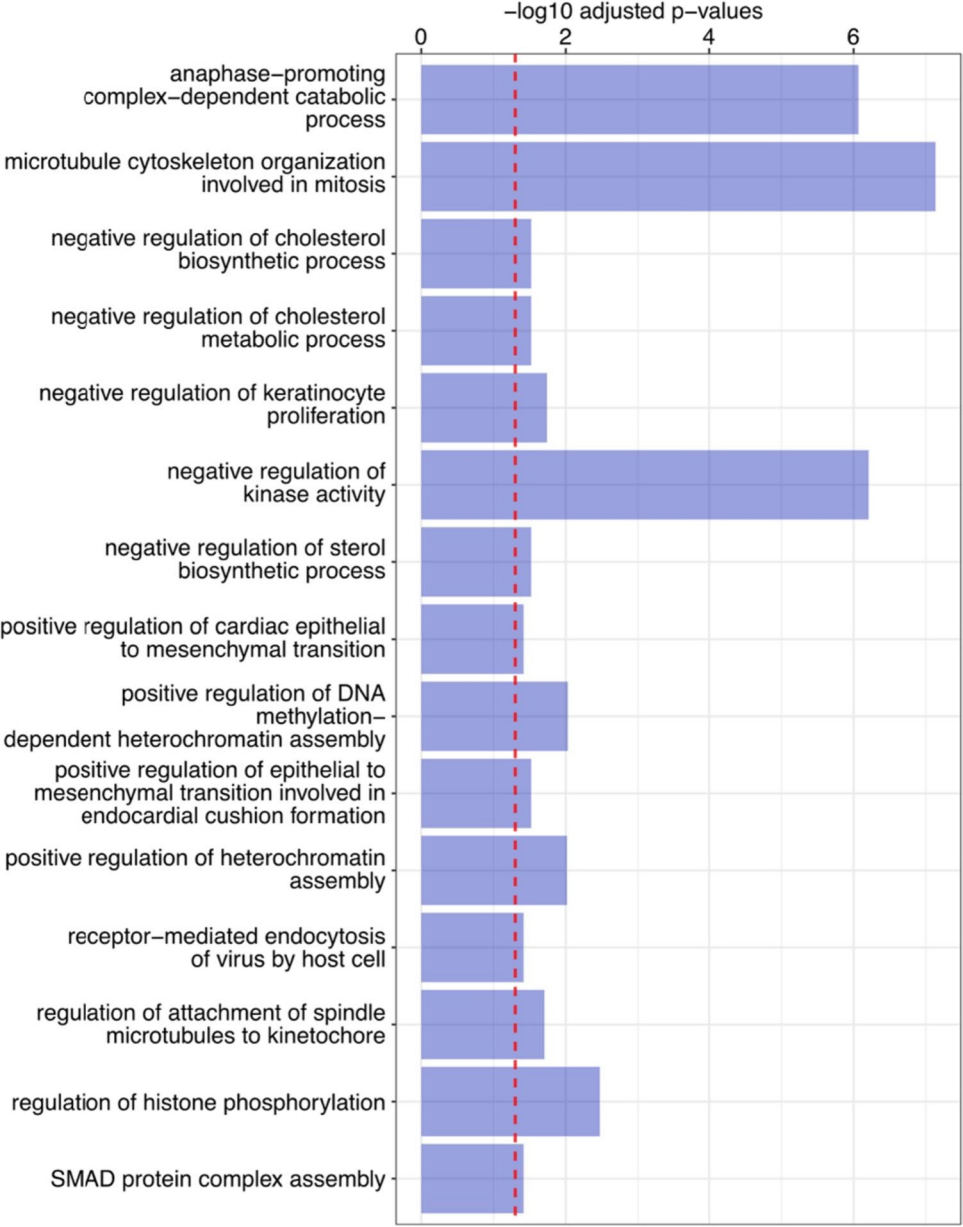
The top 15 GO terms with the highest Enrichr scores. The score signifies the level of term significance as represented by the *p* value and incorporates the *z* score, illuminating the deviation from a rank pre-established by a permutation test. Consequently, this score provides a more reliable understanding of the term's significance by factoring in the weight of the contributing genes. The dotted red line represents an adjusted *p* value of 0.05. The *y*-axis displays the -log10 transformed adjusted *p* values of the terms, derived from the identical Enrichr results

Finally, we presented 15 significant Gene Ontology Biology Process (GO BP) terms that exhibited the highest combined scores between sample subset I (*n* = 4) and II (*n* = 15) (Fig. 6). The combined scores were computed using Enrichr as previously described [17, 18].

Immunosuppressive therapy may be a potential confounding factor. Because 2 patients out of 14 in the lower score group (< 9) did not receive immunosuppressive drugs (including steroids), we performed the same analysis excluding both samples. However, PCA showed very similar results (Supplementary Fig. 2). The adjusted *p* values for DEGs differed only slightly, e.g., for *CDH1* from 0.0003665 to 0.0009462, *TLR4* from 0.001301 to 0.004026, *CXCL8* from 0.01482 to 0.01876. Due to the small cohort size, we decided not to exclude these samples and not to further stratify the groups.

We also analyzed RNA-seq data comparing COVID-19 cases assigned by clinical judgment to severe and moderate disease without considering WHO scores (Supplementary Table 6). The unsupervised PCA revealed large overlaps of HBECs treated with moderate and severe COVID-19 serum (Supplementary Fig. 3). There were no significant differences in gene expression between moderate and severe subgroups (Supplementary Fig. 4).

## Discussion

Human airway epithelial cultures are used to model chronic obstructive pulmonary disease, cystic fibrosis, and respiratory infectious diseases [19, 20]. Primary HBEC as monolayers (2-D cultures) or as 3-D cultures are good models for studying various inflammatory aspects of the lower airways [21, 22]. In this study, we applied serum from clinically and biochemically well-characterized patients infected with SARS-CoV-2 in 2D cultures of HBEC.

Severity and poor outcomes in SARS-CoV-2 infection have been associated with high levels of inflammatory mediators [23], particularly pro-inflammatory cytokines/chemokines (IL-1, IL-8, IL-12, IL-17, interferon-γ-inducible protein (IP10), MCP-1, MIP-1, and TNFα) [24, 25]. In parallel, adhesion molecules, early markers of endothelial activation/dysfunction, such as selectins (E-, P-, and L-selectin), soluble intercellular adhesion molecule 1 (ICAM-1), and vascular adhesion molecule 1 (VCAM-1), are also elevated in plasma samples from COVID-19 patients [26, 27]. Indeed, high expression of endothelial cell adhesion molecules might contribute to coagulation dysfunction [28] because these molecules are required for platelet and leukocyte migration and pro-inflammatory cytokine/chemokine production [29, 30]. Increased neutrophil counts and decreased lymphocyte counts, high levels of pro-calcitonin and D-dimer together with old age and the presence of coronary heart disease may also be useful indicators of the severity of COVID-19 disease [23, 31].

Regardless of clinical outcome, patients in our COVID-19 cohort showed limited differences in most serum inflammatory markers, although the mean or median values of several markers, such as TNFα, ICAM-1, CRP, AAT, and ferritin, were higher in more severe patients. According to previous studies, patients with severe COVID-19 have high serum concentrations of chemokines [32]. In line, E-selectin, IL-8, and MCP-1 levels were significantly higher, while albumin levels were lower in the most severe COVID-19 patients. Systematic reviews and meta-analyses have shown that serum albumin concentrations are significantly lower in COVID-19 patients with higher disease severity [33, 34]. The hypoalbuminemia is associated with severe inflammatory diseases and increased mortality [35, 36].

To make a comparative assessment of COVID-19 patients and predict clinical outcomes, several scoring systems are being implemented in clinical centers [37, 38]. Some scoring systems developed before the pandemic are also being used, such as APACHE II, which has shown promising results in predicting in-hospital patient mortality [39]. Chest X-ray (CXR), and CT scans have been proposed to predict the severity of COVID-19 by indicating the lung involvement score [40]. Another assessment model, COVID-19 BURDEN, based on the clinical features and laboratory data is available at the patient’s admission to the hospital. This model appears to improve the early detection of patients who are at a high risk of developing severe disease [41]. Among the best-validated models are those developed by Clift et al. and Knight et al. [42, 43]. The strongest predictors in these models are patient age, available clinical characteristics, treatments, and laboratory values. Recent studies have shown that machine-learning models can predict clinical severity based on commonly collected clinical data from the first 24 h of hospital admission [44].

Our patient cohort was scored according to the WHO Clinical Progression Scale (WHO-CPS), which ranks 0–10 patient illnesses by tracking progress through the health-care system [12]. Based on the WHO grading system, 5 out of 19 COVID-19 patients had the highest 9 score, i.e., ICU admission and risk of death (3 patients died). The remaining 14 patients had lower scores (between 1 and 7) and better predictive scores (only one patient died). As reflected by the WHO-CPS scores, the serum levels of some of the inflammatory markers were also higher in patients with severe disease. Therefore, based on WHO-CPS scores, we asked whether serum from COVID-19 patients with the highest or lower scores differs in their effects on the HBEC transcriptome. For this, we used a 2D HBEC culture, in which we applied 2% patient serum and incubated the cells for 18 h.

IL-8 is a potent chemoattractant and activator of immune cells and its production in epithelial cells is induced by cytokines, growth factors, bacterial and viral products, oxidants, and other factors. Accordingly, serum from patients with WHO-CPS = 9 (*n* = 5) induced more pronounced IL-8 secretion than serum from patients with WHO-CPS below 9 (*n* = 14). This result is consistent with the notion that IL-8 expression and release from HBEC correlate with disease activity [45, 46]. Next, we found that HBEC exposed to serum from 5 patients with score = 9 showed significantly lower *ACE2*, *CDH1*, and *HMOX1* expression than cells exposed to 14 patient serum with lower scores. In general, expression of *ACE2* was very low which is in line with previous studies showing that *ACE2* is highly expressed in nasal epithelial cells but much less in HBEC [47]. According to other studies, higher *ACE2* levels have a protective effect against COVID-19 and associated complications, particularly cardiac adverse events [48, 49]. In fact, the expression of ACE2 is decreased post viral entry [50]. The *CDH1* gene encodes E-cadherin, a transmembrane calcium-dependent adhesion molecule expressed in epithelial cells [51]. CDH1 has been identified as a potential regulator of epithelial barrier function [52], showing lower expression in SARS-CoV-2-infected cells [50, 53]. Studies have demonstrated that SARS-CoV-2 infection also decreases the expression of antioxidant genes, such as *HMOX1* [54]. The HMOX1 pathway can inhibit platelet aggregation and has anti-thrombotic and anti-inflammatory properties, all of which are compromised during critical medical conditions in COVID-19 patients [55, 56]. Taken together, the decrease in the expression of *CDH1*, *ACE2,* and *HMOX1* genes suggests that HBECs exposed to serum from the most severe COVID-19 patients (WHO-CPS = 9) acquire a dysfunctional and/or infected phenotype.

To further characterize the changes that occur in HBEC exposed to the serum of COVID-19 patients, we profiled the cell transcriptomics. The core-expression signature based on 500 genes segregated cells into two subsets: those exposed to the serum of four (subset I) and 15 (subset II) patients. It is important to note that all four patients in cell subset I had a WHO-CPS of 9, while one patient with a WHO-CPS of 9 occurred in cell subset II. Unfortunately, by reviewing all available clinical and laboratory data for the latter patient, we were unable to explain this discrepancy.

The cells exposed to serum from four patients (subset I) showed 5566 DEGs compared to cells treated with serum from subset II patients (*n* = 15). Among other genes, higher expression of *TLR4* and *CXCL8* but lower *CDH1* was found in cell subset I than in subset II. In general, these four serum samples had the strongest effects on genes involved in metabolic regulation, cytoskeleton organization, and kinase activity pathways. Notably, changes in kinase activity are typically associated with viral infection, and kinases represent ideal drug targets.

Our study has a few limitations. First, we had a small patient cohort and serum effects were investigated in a HBEC monolayer culture derived from a single healthy donor. In recent years, studies prefer to use various bronchial cell air–liquid interface culture (ALI) protocols to generate differentiated monocultures of bronchial epithelium. However, ALI models also have limitations, such as that complex differentiation protocols harbor problems with the reproducibility, the growth surface is much stiffer than the in vivo tissue microenvironment; ALIs lack cell–cell interactions with non-epithelial cells and the extracellular matrix [57]. These latter affect the HBE phenotype, heterogeneity, and functionality in vitro. The morphological structure and heterogeneity of the ALI epithelium is also affected by the collection sites and techniques used for collecting donor cells, and by donor-specific variations. Therefore, accessible and easy-to-handle HBEC-based model may be useful to complement 2D ALIs as well as novel 3D organ tissue equivalent (OTE) airway models [58], and help to obtain initial data on the putative serum activity.

Next, human respiratory epithelium is the main target of viral infections and acts as an innate immune sensor during infections, it expresses pattern recognition receptors, pro-inflammatory cytokines, chemokines, and growth factors [59]. The interactions between viral infection-induced inflammatory molecules and the apical epithelial surface-initiated secretion of cytokines and chemokines from these cells lead to leukocyte recruitment and escalation of inflammatory responses. The specific signaling pathways that play a role in this scenario are not fully understood. We are aware that inflammatory molecules are not the same qualitatively and quantitatively in the peripheral blood and locally in the airways. However, from critical-ill patients, it is often not possible to obtain local fluids for experimental studies. The choice of serum was also because the shedding of blood proteins actively contributes to the formation of a pro-inflammatory environment. Therefore, results from a simple blood serum test model using HEBC can be valuable in the development of more complex analyses/models in clinical research. For example, HBECs are SARS-CoV-2 target cells as they express ACE2 that is used by SARS-CoV-2 as a receptor for entry and the proteases TMPRSS2 and cathepsin L for priming the S protein. The SARS-CoV-2 infected epithelial and ciliated airway cells potentiate immune cell activation and systemic hyper-inflammatory state of COVID-19 patient [60]. Studies suggest that strong immune response characterized by cytokine/chemokine storm rather than direct virus-induced damage is responsible for COVID-19 pathogenesis [61]. In line, we demonstrate that serum molecules from clinically well-characterized COVID-19 patients reflect bystander effect of SARS-CoV-2 on HBECs. Serum from most severe COVID-19 patients (WHO-CPS = 9) gave stronger effects on genes involved in metabolic regulation, and cytoskeleton organization pathways. Results from NHBE cells infected with SARS-CoV also found dysregulation in cytoskeleton-related genes [62]. Other investigators reported that treatment of HBEC with serum from COPD patients increased expression of senescence markers and the secretion of IL-8, CXCL5, and VEGF-A relative to serum from healthy controls [63]. These studies further support an idea that serum-induced alterations in HBEC reflect inflammatory status.

## Conclusion

We demonstrate that HBEC may be a simple model useful to validate the bronchial epithelial responses to serum biomarkers related to the inflammatory state caused by COVID-19 or other infections. Although numerous data support the systemic inflammatory component in patients with respiratory diseases, it is often not possible to predict disease development based on one or a few specific inflammatory factors per se. Serum inflammation-based scores can be useful in predicting disease severity and outcomes; however, some patients may have unknown/unreported diseases, as well as infections, influencing markers of systemic inflammation. On the other hand, many serum markers are not routinely measured in clinical laboratories and are not studied or not used or in clinical practice. Therefore, 2D models of epithelial cells can be useful for characterizing the serum of patients with different degrees of inflammation and disease severity. Longitudinal samples from patients would be useful for correlating the clinical course with changes in HBEC transcriptional properties.

### Supplementary Information

Below is the link to the electronic supplementary material.

Supplementary file1 (PDF 549 KB)
