# Supplementary material for: Human Bronchial Epithelial Cell Transcriptome Changes in Response to Serum from Patients with Different Status of Inflammation
Source: Lung. 2024 Mar 17;202(2):157–70. doi: 10.1007/s00408-024-00679-1 (PMC11009779; doi:10.1007/s00408-024-00679-1)
Supplement: Supplementary file 1 — Supplementary file1 (PDF 549 KB) [file 408_2024_679_MOESM1_ESM.pdf]

Supplementary Table 1: Serum levels of cytokines/chemokines

Serum Marker*	WHO-CPS = 9	N	WHO-CPS < 9	N	p-value
CD62P (ng/ml)	140.9 (139.0)	5	159.2 (121.8)	14	0.7840
ICAM-1 (µg/ml)	1.730 (0.902-3.155)	5	0.632 (0.405-1.480)	14	0.0700
IFN-α (pg/ml)	2.72 (2.22)	3	4.49 (1.58)	7	0.1828
IL-1α (pg/ml)	3.96 (1.10-5.12)	5	1.50 (1.16-4.15)	13	0.1670
IL-12 P70 (pg/ml)	39.9 (33.8-79.7)	5	36.6 (33.8-48.1)	14	0.4044
IL-17α (pg/ml)	23.8 (21.9-47.9)	5	19.7 (14.2-37.0)	14	0.2274
IP-10 (pg/ml)	110.3 (71.0-379.4)	5	156.0 (48.6-310.1)	14	0.8932
MIP-1α (pg/ml)	19.2 (13.0)	4	24.9 (19.0)	5	0.6269
MIP-1β (pg/ml)	119.4 (63.3-403.8)	5	71.1 (43.2-112.4)	12	0.3284
TNF-α (pg/ml)	66.3 (50.5-111.9)	5	57.5 (46.3-79.1)	14	0.7009

All analyses were carried out in duplicates. If Shapiro-Wilk normality test passed, data are shown as mean (SD) and p-values were calculated with two-sided unpaired t test. If normality test failed, data are presented as median (IQR) and p-values are calculated with Mann-Whitney test. A p-value below 0.05 was considered as significant.

Supplementary Table 2: Effect of patient serum on specific genes in HBEC relative to *POLR2A*, a housekeeping gene.

Gene	WHO-CPS = 9	N	WHO-CPS < 9	N	p-Value
CXCL8	11.79 (4.52)	5	9.05 (3.64)	14	0.1908
FITM2	0.057 (0.016)	5	0.063 (0.020)	14	0.5799
FITM1	0.0008 (0.0006-0.0012)	5	0.0011 (0.0008-0.0013)	14	0.2566
IL1A	24.65 (4.60)	5	23.6 (5.11)	14	0.6768
*PECAM1**	0.0003	2	0.0007 (0.0005-0.0009)	10	-
SERPINA	0.0084 (0.0065-0.0092)	5	0.0094 (0.0083-0.0104)	14	0.0870
SERPINE	18.17 (11.47-34.84)	5	24.62 (16.36-31.79)	14	0.3426
TLR2	0.034 (0.016)	4	0.028 (0.011)	13	0.4024
TMPRSS2	0.011 (0.005)	5	0.014 (0.006)	14	0.3664
TNF	0.023 (0.022-0.038)	5	0.022 (0.017-0.033)	14	0.5593
VEGFA	8.05 (1.15)	5	7.51 (1.49)	14	0.5338

CXCL8, C-X-C motif chemokine ligand 8 (IL-8); *FITM*, fat storage-inducing transmembrane protein; *IL1A*, interleukin-1α; *PECAM1*, platelet endothelial cell adhesion molecule 1; *TLR*, toll-like receptor; *TMPRSS2*, transmembrane protease serine subtype 2; *TNF*, tumor necrosis factor; *VEGFA*, vascular endothelial growth factor A; CPS, clinical progression scale. *Missing samples were below the detection range. All analyses were performed in duplicates. If the Shapiro-Wilk normality test was passed, data were shown as mean (SD), and p-values were calculated using a two-sided unpaired t-test. If the normality test failed, the data were presented as median (IQR), and p-values were calculated using the Mann-Whitney test. Statistical significance was set at $p < 0.05$.

Supplementary Table 3: Cohort description of 4 specific cases and general COVID-19 cases

Variables	4 specific cases	general COVID	p-value
**Groups, n (%)**	4 (21)	15 (78.9)	
**Age, mean (SD)**	61.8 (3.3)	61.5 (19.9)	0.9833
**Gender (female / male)**	1 / 3	7 / 8	
**BMI**	34 (27-43)	28 (27-32)	0.4773
**Vaccination status (Yes / No, n / n)**	0 / 4	0 / 15	
**Death due to COVID-19, n (%)**	2 (50.0)	2 (10.5)	
**Comorbidities (Yes / No / Unkown, n / n / n)**			
Lung disease	0 / 2 / 2	0 / 10 / 5	
Diabetes	0 / 3 / 1	2 / 8 / 5	
Heart disease	0 / 1 / 3	2 / 7 / 6	
Adiposity	2 / 1 / 1	2 / 6 / 7	
Arterial hypertension	3 / 1 / 0	6 / 8 / 1	
Heart disease	0 / 1 / 3	2 / 7 / 6	
Kidney disaese	0 / 4 / 0	4 / 10 / 1	
Liver disease	2 / 2 / 0	1 / 13 / 1	
Immunological diseases (Vasculitis, diverticulitis)	0 / 4 / 0	2 / 13 / 0	
Pregnancy	0 / 4 / 0	3 / 12 / 0	
Organ transplantation	0 / 4 / 0	1 / 14 / 0	
Active tumor (Yes / No / in remission)	0 / 4 / 0	2 / 11 / 2	
**Chronic therapy (Yes / No, n / n)**			
Cortisone	0 / 4	2 / 13	
Immunosuppressive drugs	0 / 4	3 / 12	
**Place of birth (Europe / Other / Unkown, n / n / n)**	3 / 1 / 0	9 / 5 / 1	
**Smoking status (Active / Never- / Ex- / Unkown, n / n / n / n)**	0 / 0 / 0 / 4	1 / 6 / 3 / 5	
**Day of sampling (Known / Unknown)**	3 / 1	8 / 7	
Disease day, mean (SD)	15.0 (5.0)	11.0 (6.4)	0.3615
**Acute COVID-19 therapy**			
ICU, n (%)	4 (21.1)	6 (32)	
Mechanical ventilation, n (%)	4 (21.1)	2 (10.5)	
ECMO, n (%)	3 (15.9)	1 (5.3)	
Vasopressors, n(%)	4 (21.1)	1 (5.3)	
Oxygen by NIV or high flow, n (%)	0 (0.0)	4 (21.1)	
Oxygen by mask or nasal prongs, n (%)	0 (0.0)	6 (31.6)	
Dialysis (Yes / No / Unkown, n / n / n)	2 / 2 / 0	0 / 14 / 1	
Anticoagulation (Yes / No / Unkown, n / n / n)	2 / 0 / 2	9 / 3 / 3	
Steroids, n (%)	4 (21.1)	9 (47.4)	
**Complications**			
Renal failure	2 (10.5)	4 (21.1)	
Liver failure	2 (10.5)	0 (0.0)	
ARDS	4 (21.1)	4 (21.1)	
**Clinical Parameters**			
AAT (mg/ml), n / mean (SD)	4 / 784 (182)	15 / 606 (136)	**0.0433**
Albumin (g/l), n / mean (SD)	4 / 17.8 (2.4)	8 / 29.4 (8.7)	**0.0273**
CRP (mg/l), n / median (IQR)	4 / 102 (93-198)	12 / 47 (11-122)	0.1033
D-Dimer (mg/l), n / median (IQR)	4 / 2.2 (1.4-4.6)	12 / 2.2 (1.4-6.2)	0.9335
Ferritin (µg/l), n / mean (SD)	4 / 991 (251)	12 / 596 (505)	0.1607
Hyaluronic Acid (ng/ml), n / median (IQR)	4 / 92 (60-643)	9 / 81 (28-153)	0.5304
INR (ratio), n / median (IQR)	4 / 1.1 (1.0-1,1)	12 / 0.9 (0.9-1.1)	0.2203
Lipase (U/l), n / mean (SD)	4 / 42.8 (33.5)	10 / 43.2 (31.1)	0.9813

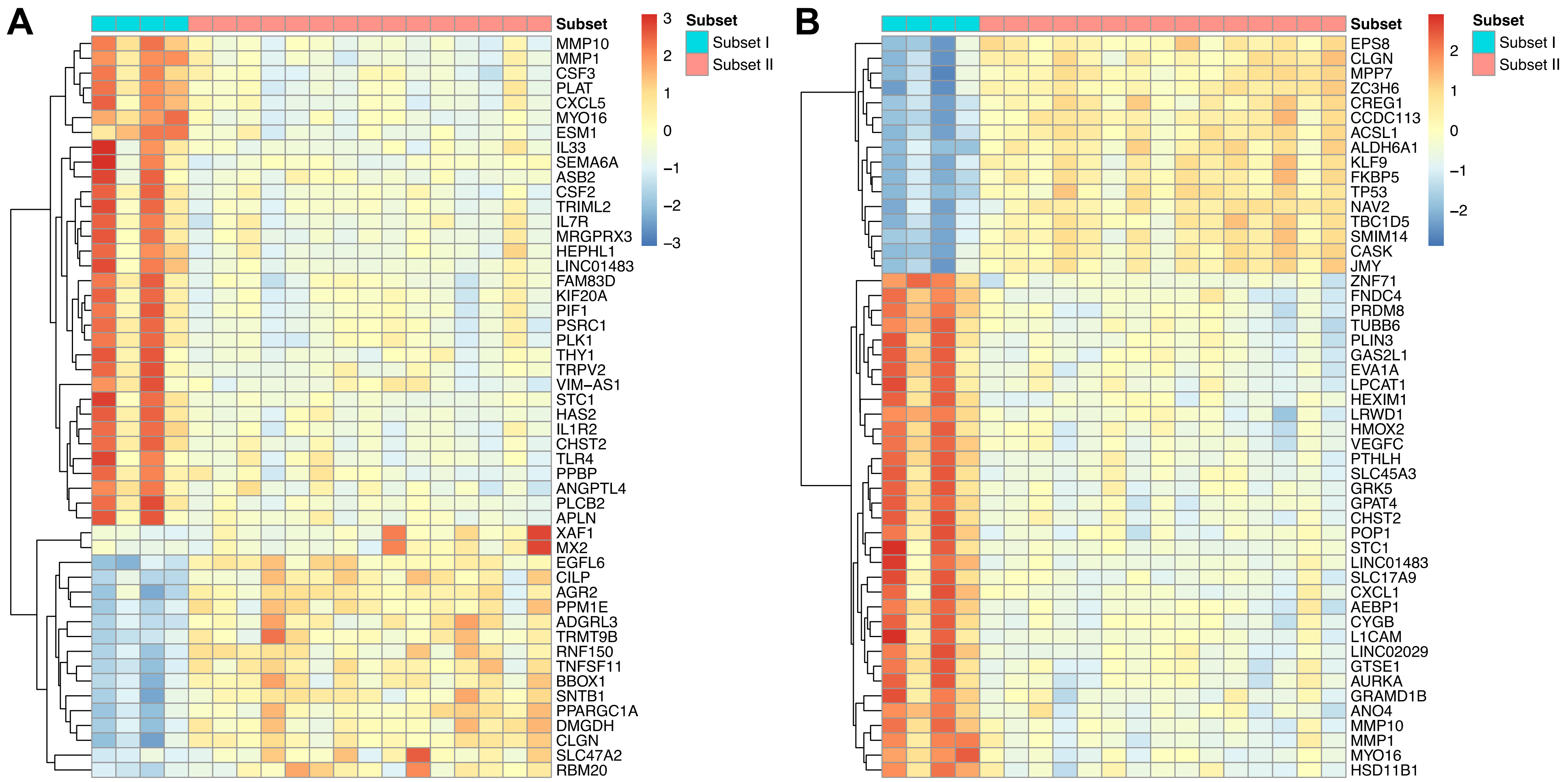

Supplementary figure 1: Visualization of differential gene expression of HBEC after culture for 18 h with medium supplemented with COVID-19 patient serum. HBEC were cultured with 2% serum from the 4 COVID-19 patients with a WHO-CPS = 9 (subset I, green), which were identified by PCA (figure 2, lightred), compared to cells cultures with a serum from 15 patients (subset II, figure 2, red and purple dots). Cell colors reflect the frequency of the gene. **A.** The top 50 DEGs out of 5566 with highest log2 fold change. **B**. The top 50 DEGs with lowest adjusted p-value.

Supplementary table 4: Gene Annotation for log2-fold change-based top 50 DEGs of HBECs cultured in presence of specific COVID-19 serum or general COVID-19 serum.

Gene Symbol	log2FC	Significant_GOs
STC1	4.072808054	Regulation of cell migration
LINC01483	3.754958924	Not Represented in GO Enrichment Analysis
HAS2	3.544921606	Positive regulation of cell migration,regulation of cell migration,positive regulation of cell motility
ADGRL3	-3.021782537	Absent in Significant GO Terms
TNFSF11	-2.916334334	Regulation of transcription by RNA polymerase II,positive regulation of transcription, DNA-templated,regulation of intracellular signal transduction
CILP	-2.873274938	Cellular response to growth factor stimulus,cellular response to transforming growth factor beta stimulus,regulation of gene expression
RNF150	-2.856453842	Ubiquitin-dependent protein catabolic process,modification-dependent protein catabolic process
IL1R2	2.773024793	Interleukin-1-mediated signaling pathway,cytokine-mediated signaling pathway
ESM1	2.754276955	Regulation of cell population proliferation,positive regulation of signal transduction
CXCL5	2.737703043	Regulation of cell population proliferation,cytokine-mediated signaling pathway
PIF1	2.72191963	Negative regulation of transferase activity,regulation of telomerase activity,regulation of DNA metabolic process
CSF2	2.548807779	Negative regulation of transcription, DNA-templated,negative regulation of cellular macromolecule biosynthetic process,negative regulation of nucleic acid-templated transcription
TRIML2	2.531456308	Protein polyubiquitination,protein ubiquitination,regulation of autophagy
CSF3	2.452756215	Regulation of transcription by RNA polymerase II,positive regulation of transcription, DNA-templated,positive regulation of transcription by RNA polymerase II
ANGPTL4	2.43750827	Regulation of apoptotic process,negative regulation of programmed cell death,negative regulation of apoptotic process
APLN	2.385743692	Positive regulation of transcription by RNA polymerase II,regulation of gene expression
KIF20A	2.36993249	Mitotic cytokinesis,regulation of cell cycle process,cytoskeleton-dependent cytokinesis
MMP1	2.288503588	Regulation of cell growth,positive regulation of cell migration,regulation of cell migration
MX2	-2.260441141	Not Represented in GO Enrichment Analysis
ASB2	2.206592418	Ubiquitin-dependent protein catabolic process,cellular protein modification process,modification-dependent protein catabolic process
TRMT9B	-2.176588035	tRNA processing
PLAT	2.152637806	Cellular protein modification process,protein modification process,transmembrane receptor protein tyrosine kinase signaling pathway
MRGPRX3	2.147037462	Not Represented in GO Enrichment Analysis
DMGDH	-2.141885428	Absent in Significant GO Terms
PPM1E	-2.106743965	Cellular protein modification process,negative regulation of kinase activity,negative regulation of protein phosphorylation
PLCB2	2.106435815	polyol metabolic process,non-canonical Wnt signaling pathway,inositol phosphate metabolic process
PLK1	2.096553933	Proteasome-mediated ubiquitin-dependent protein catabolic process,ubiquitin-dependent protein catabolic process,regulation of cell cycle
THY1	2.092649741	Negative regulation of kinase activity,negative regulation of protein phosphorylation,organelle organization
IL33	1.982052178	Negative regulation of transcription, DNA-templated,protein modification by small protein removal,protein deubiquitination
XAF1	-1.975725932	Cytokine-mediated signaling pathway
MYO16	1.96598375	Negative regulation of mitotic cell cycle phase transition,negative regulation of cellular process,regulation of cell population proliferation
TLR4	1.959958179	Regulation of transcription by RNA polymerase II,positive regulation of transcription, DNA-templated,positive regulation of transcription by RNA polymerase II
CHST2	1.948186069	Absent in Significant GO Terms
AGR2	-1.939435624	Negative regulation of cellular process,regulation of gene expression,positive regulation of growth
PPARGC1A	-1.90185232	Regulation of transcription by RNA polymerase II,organelle organization,regulation of transcription, DNA-templated
HEPHL1	1.899410059	Absent in Significant GO Terms
TRPV2	1.892900725	Absent in Significant GO Terms
SLC47A2	-1.872116595	Not Represented in GO Enrichment Analysis

EGFL6	-1.872077602	Not Represented in GO Enrichment Analysis
MMP10	1.83814301	Absent in Significant GO Terms
SNTB1	-1.82827526	Absent in Significant GO Terms
CLGN	-1.819920939	Not Represented in GO Enrichment Analysis
SEMA6A	1.804106912	Organelle organization,positive regulation of cell migration,regulation of cell migration
IL7R	1.795863726	Regulation of cell population proliferation,membrane organization,cytokine-mediated signaling pathway
RBM20	-1.784642803	Regulation of gene expression
BBOX1	-1.780975849	Absent in Significant GO Terms
FAM83D	1.780417155	Regulation of intracellular signal transduction,mesenchymal cell differentiation,epithelial to mesenchymal transition
PSRC1	1.772614296	Mitotic sister chromatid segregation,regulation of cell growth,regulation of transcription, DNA-templated
PPBP	1.772599688	Proteasome-mediated ubiquitin-dependent protein catabolic process,ubiquitin-dependent protein catabolic process,proteasomal protein catabolic process
VIM-AS1	1.770021211	Not Represented in GO Enrichment Analysis

Supplementary table 5: Top 50 DEGs related pathways

nGenes	Pathway	Genes
5	Rheumatoid arthritis	*CSF2, MMP1, CXCL5, TLR4, TNFSF11*
4	IL-17 signaling pathway	*CSF2, CSF3, MMP1, CXCL5*
4	Hematopoietic cell lineage	*CSF2, CSF3, IL7R, IL1R2*
4	Amoebiasis	*CSF2, PLCB2, TLR4, IL1R2*
4	Apelin signaling pathway	*PPARGC1A, PLAT, PLCB2, APLN*
8	Cytokine-cytokine receptor interaction	*CSF2, CSF3, IL7R, PPBP, CXCL5, IL1R2, TNFSF11, IL33*
5	Coronavirus disease-COVID-19	*CSF2, CSF3, MMP1, MX2, TLR4*

Gene ontololgy enrichment software: http://bioinformatics.sdstate.edu/go/

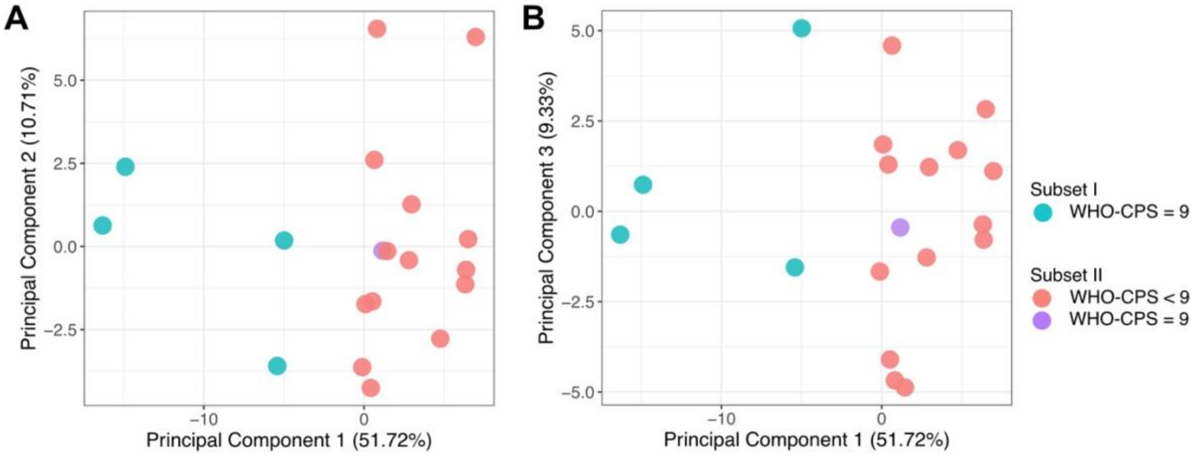

Supplementary figure 2: Principal component analysis of RNA-seq data from HBEC treated with 2% of patient serum excluding 2 patients not receiving immunosuppressive medication. PCA on top 500 transcripts revealed a subset I of four clearly distinguishable patients all of which had a WHO-CPS of 9 (labeled in blue). A subset II comprises 14 samples with a WHO-CPS of 1 to 7 (labeled in red) and one outlier sample with WHO-CPS = 9 (labeled in purple). The x- and y-axis represent the first (A) and second (B) principal components (PCs), respectively. The first PCA distinctly segregates the patients into two subgroups, accounting for 51.72% of the total variance among the samples.

Supplementary Table 6. Cohort description of severe vs moderate COVID-19 cases

Variables	Severe	Moderate	p-value
**Groups, n (%)**	10 (52.6)	9 (47.4)	
**Age median, (IQR)**	53.9 (16.1)	70.1 (15.9)	**0.0415**
**Gender (female / male)**	5 / 5	3 / 6	
**Death due to COVID-19, n (%)**	4 (21.1)	0 (0.0)	
**Comorbidities (Yes / No / Unkown, n / n / n)**			
Lung disease	0 / 5 / 5	0 / 7 / 2	
Diabetes	0 / 10 / 0	2 / 7 / 0	
Heart disease	0 / 4 / 6	2 / 4 / 3	
Adiposity	5 / 4 / 1	3 / 5 / 1	
Arterial hypertension	6 / 4 / 0	3 / 6 / 0	
Heart disease	1 / 9 / 0	2 / 7 / 0	
Kidney disaese	2 / 7 / 1	2 / 7 / 0	
Liver disease	2 / 7 / 1	1 / 8 / 0	
Immunological diseases (Vasculitis, diverticulitis)	0 / 10 / 0	2 / 7 / 0	
Pregnancy	2 / 8 / 0	1 / 8 / 0	
Organ transplantation	1 / 0 / 0	0 / 0 / 0	
Active tumor (Yes / No / in remission)	0 / 9 / 1	2 / 7 / 1	
**Chronic therapy (Yes / No, n / n)**			
Cortisone	1 / 9	1 / 8	
Immunosuppressive drugs	2 / 8	1 / 8	
**Place of birth (Europe / Other / Unkown, n / n / n)**	6 / 4 / 0	6 / 2 / 1	
**Smoking status (Active / Never- / Ex- / Unkown, n / n / n / n)**	0 / 3 / 1 / 6	1 / 3 / 2 / 3	
**Day of sampling (Known / Unknown)**	6 / 4	5 / 4	
Disease day, Mean (SD)	13.7 (5.4)	10.2 (7.1)	0.3774
**Acute COVID-19 therapy**			
ICU, n (%)	10 (52.6)	0 (0.0)	
Mechanical ventilation, n (%)	6 (31.6)	0 (0.0)	
ECMO, n (%)	3 (15.8)	0 (0.0)	
Oxygen by NIV or high flow, n (%)	4 (21.1)	0 (0.0)	
Oxygen by mask or nasal prongs, n (%)	0 (0.0)	6 (31.6)	
Dialysis (Yes / No / Unkown, n / n / n)	2 / 8 / 0	0 / 8 / 1	
Anticoagulation (Yes / No / Unkown, n / n / n)	8 / 0 / 2	3 / 4 / 2	
Steroids, n (%)	10 (52.6)	6 (31.6)	
**Clinical Parameters**			
AAT (mg/ml), n / mean (SD)	10 / 692 (139)	9 / 589 (171)	0.1700
Albumin (g/l), n / mean (SD)	10 / 23.3 (8.2)	2 / 36.5 (3.5)	n. a.
CRP (mg/l), n / mean (SD)	10 / 104.5 (80.4)	6 / 50.5 (59.9)	0.1781
D-Dimer (mg/l), n / median (IQR)	10 / 2.2 (1.5-5.9)	6 / 2.2 (1.1-5.6)	0.8961
Ferritin (µg/l), n / mean (SD)	10 / 689 (444)	6 / 704 (581)	0.9538
Hyaluronic Acid (ng/ml), n / median (IQR)	10 / 99 (52-184)	9 / 70.6 (24-135)	0.4002
INR (ratio), n / median (IQR)	10 / 1.0 (0.9-1,2)	6 / 0.9 (0.8-1.3)	0.3524
Lipase (U/l), n / mean (SD)	9 / 44.0 (35.7)	5 / 41.4 (21.5)	0.8855

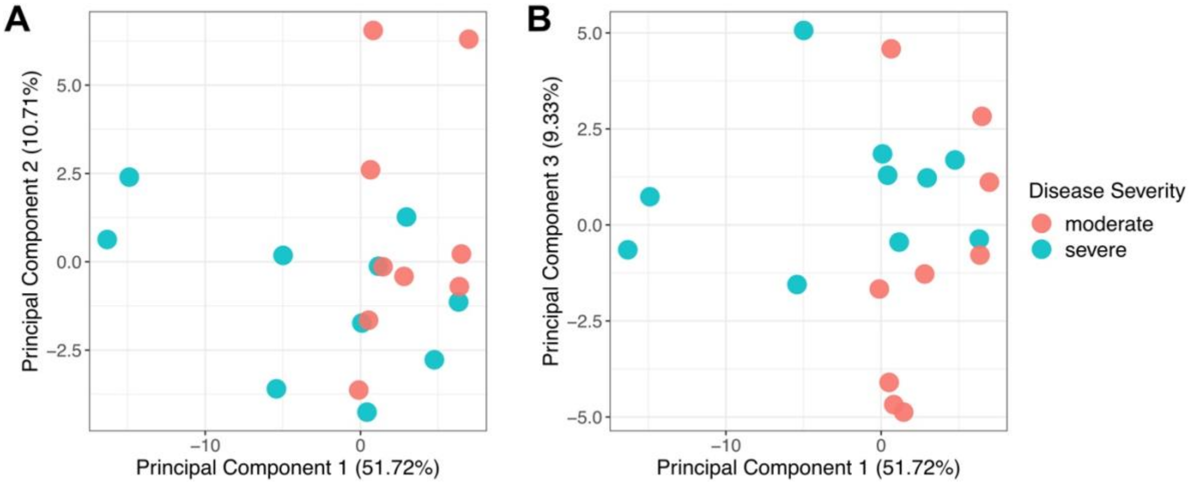

Supplementary figure 3: Principal component analysis of RNA-seq data from HBEC cultured with 2% serum from patients with severe COVID-19 compared to cells cultured with moderate COVID-19 serum. The x- and y-axis represent the first (A) and second (B) principal components (PCs), respectively. Both charts show overlaps between the different severity groups.

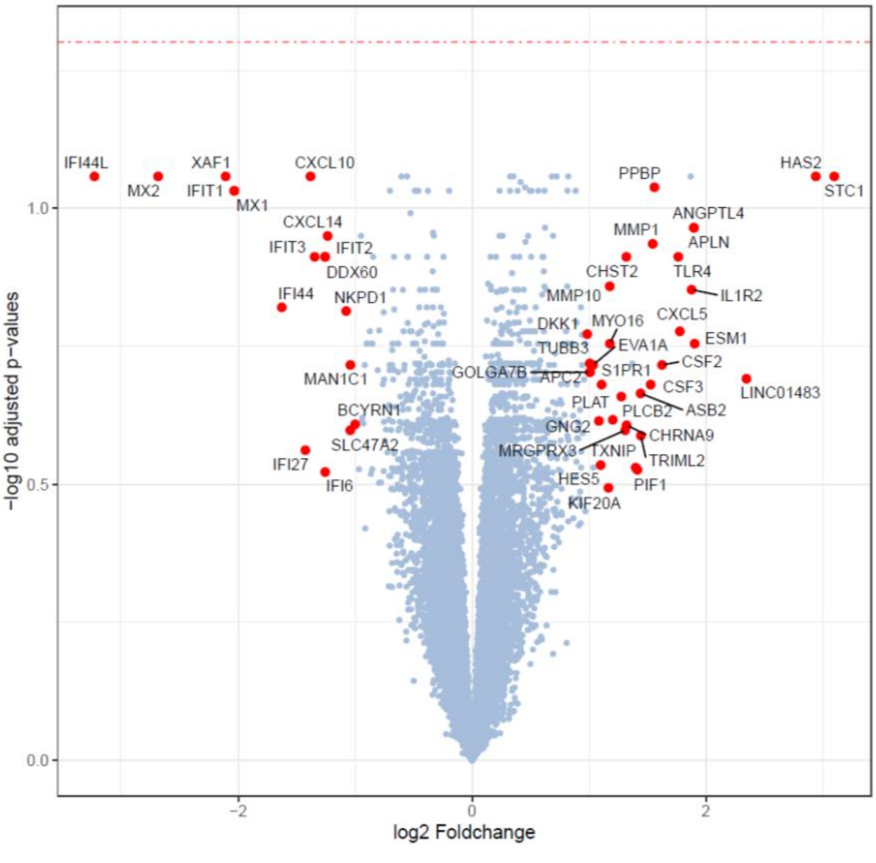

Supplementary Figure 4: The volcano plot shows the differential gene expression analysis (DEA) of HBEC cultured with 2% serum from patients with severe COVID-19 compared to cells cultured with moderate COVID-19 serum Selected DEGs are labeled. The red dotted line marks a threshold of significance, corresponding to an adjusted p-value of 0.05. None of the DEGs is significant.
